# Supplementary material for: Recombinant production of spider silk protein in Physcomitrella photobioreactors
Source: Plant Cell Rep. 2025 Apr 26;44(5):103. doi: 10.1007/s00299-025-03485-y (PMC12033203; doi:10.1007/s00299-025-03485-y)
Supplement: Supplementary file 1 — Supplementary file1 (DOCX 8691 KB) [file 299_2025_3485_MOESM1_ESM.docx]

**Supplemental Information**

**Recombinant production of spider silk protein in Physcomitrella photobioreactors**

**
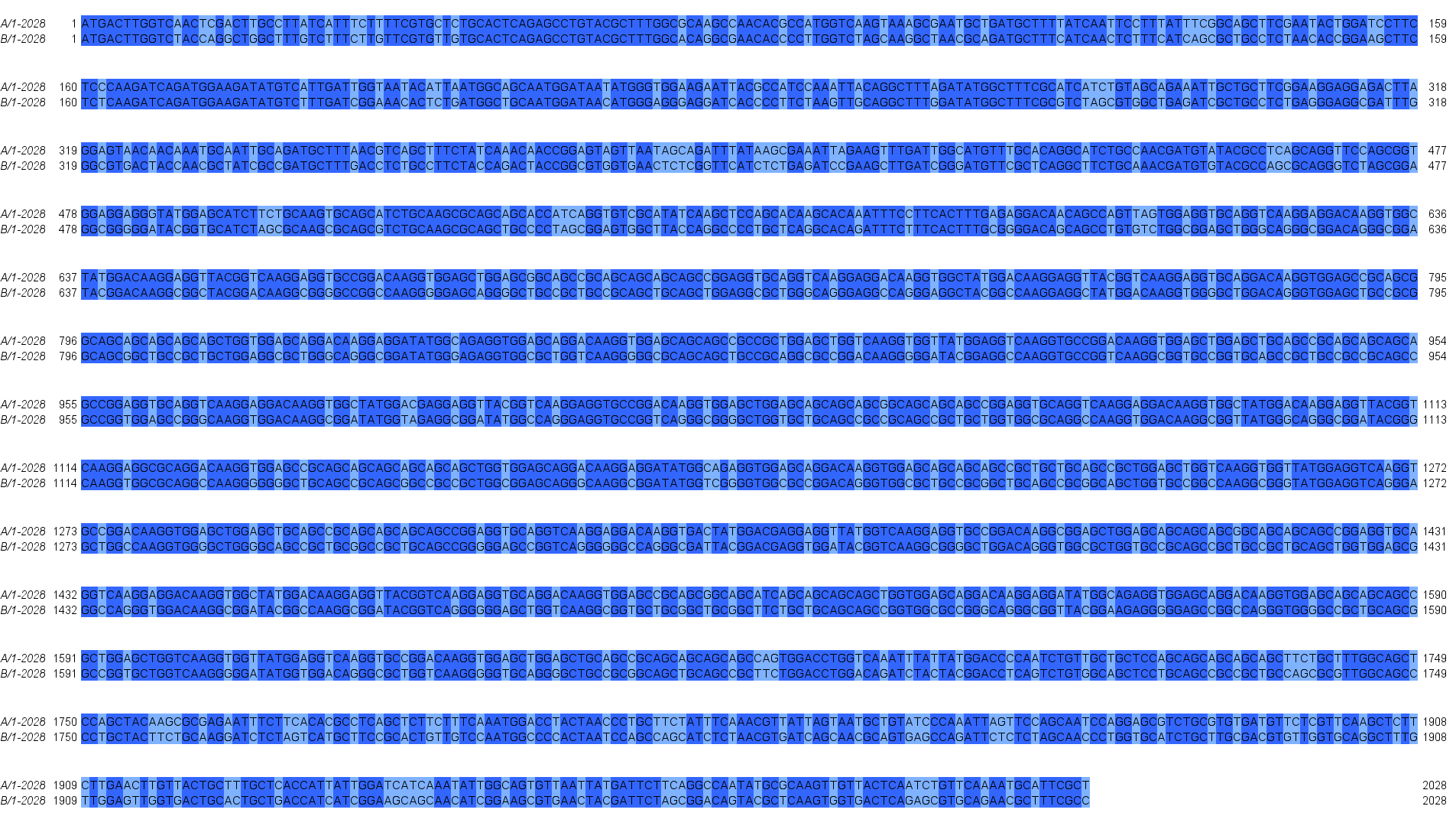
**

**Fig. S1 Pairwise sequence alignment of the original NTD-LhMaSp1-12Rep-CTD (A), and the codon optimized NTD-LhMaSp1-12Rep-CTD for codon usage in Physcomitrella (B).** Alignment was performed with Jalview (Version 2.11.3.3, Waterhouse et al. 2009)

**
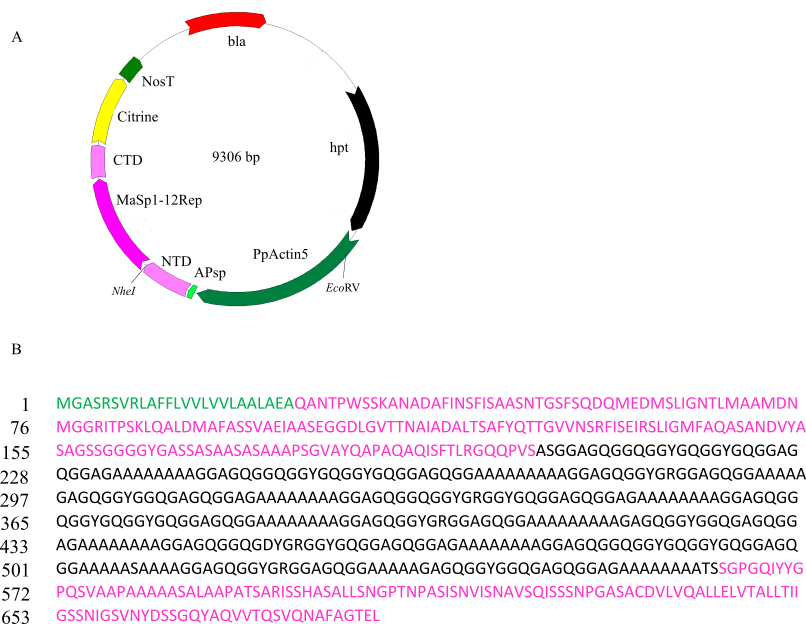
**

**Fig. S2 Designed construct for the transient expression of NTD-LhMaSp1-12Rep-CTD-citrine in Physcomitrella protoplasts. A** Plasmid used for monitoring the localisation of MaSp1-12Rep into the secretory pathway of Physcomitrella. This plasmid includes the coding sequence of PpActin5 promoter, APsp, 12Rep, NTD and CTD, citrine Fluorescence protein, Nos terminator, and bla (beta-lactamase). **B** Protein sequence of NTD-LhMaSp1-12Rep-CTD. Green: APsp. Black: 12 repetitive Physcomitrella codon-optimized poly-alanine blocks of *L. hesperus* silk protein core region (12Rep). Purple: the full sequence of N and C-terminal domain (NTD and CTD).


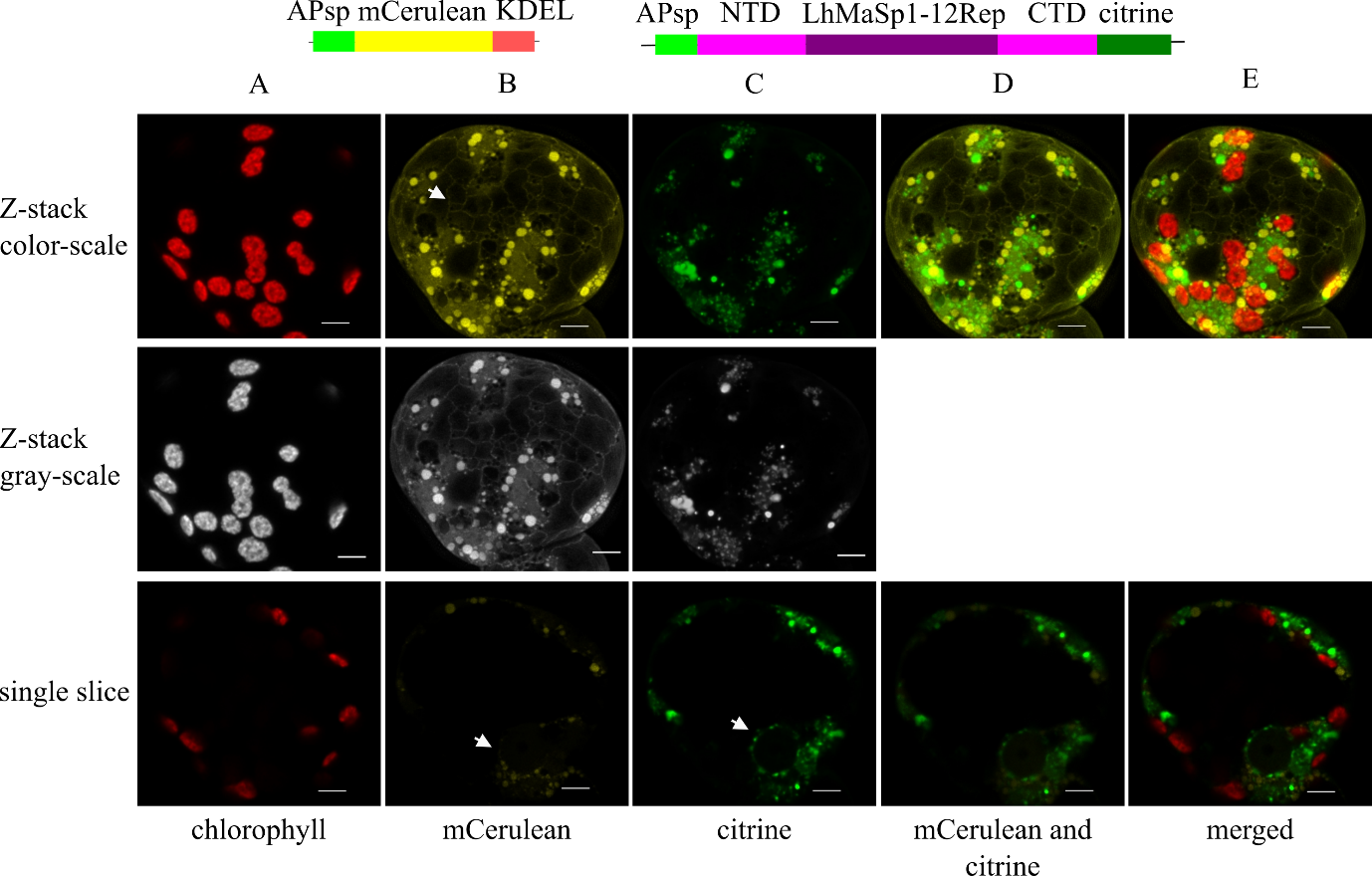


**Fig. S3 Confocal microscopy images showing the localisation of NTD-LhMaSp1-12Rep-CTD fused to citrine in Physcomitrella protoplasts**. Schematic representation of the fusion proteins is at the top. The mCerulean-KDEL construct was used as positive control for ER localisation. **A** Chlorophyll autofluorescence. **B** The arrows show the fluorescence signal of ramified network of mCerulean-KDEL and its localisation to the nuclear envelope. **C** The arrow indicates the presence of NTD-LhMaSp1-12Rep-CTD protein fused to citrine in the nuclear envelope. The ramified network is not observed. **D** Citrine and mCerulean signals do not overlap, but cover almost the same area. **E** merged channels. The upper images display 3D-rendered Z-stack images, while the lower ones depict individual slices of a cell. Grey-scale channels are provided for better visibility of images. Bars = 5 µm.


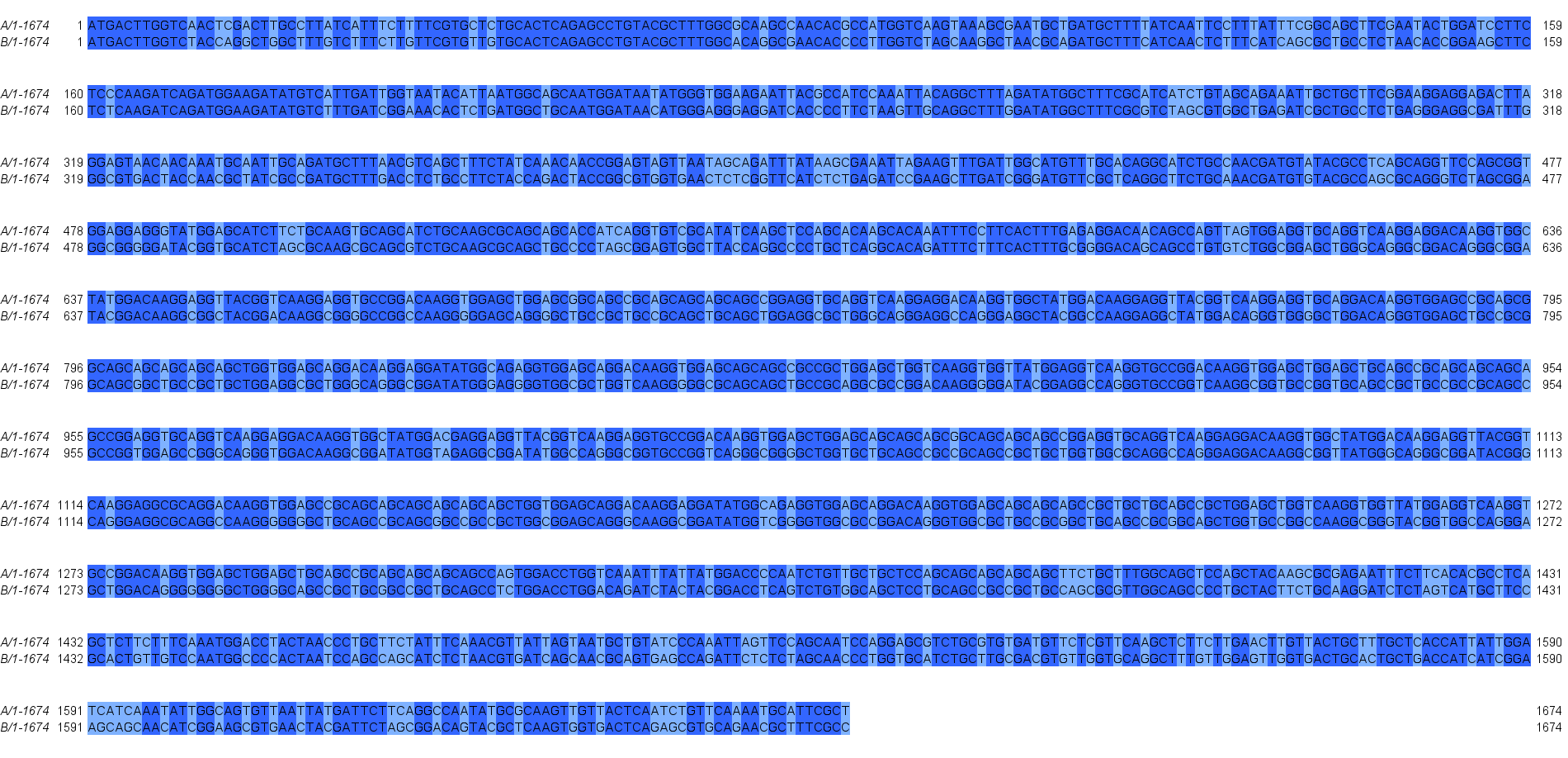


**Fig. S4 Pairwise sequence alignment of the original NTD-LhMaSp1-8Rep-CTD (A), and the codon optimized NTD-LhMaSp1-8Rep-CTD for codon usage in Physcomitrella (B).** Alignment was performed with Jalview (Version 2.11.3.3, Waterhouse et al. 2009)


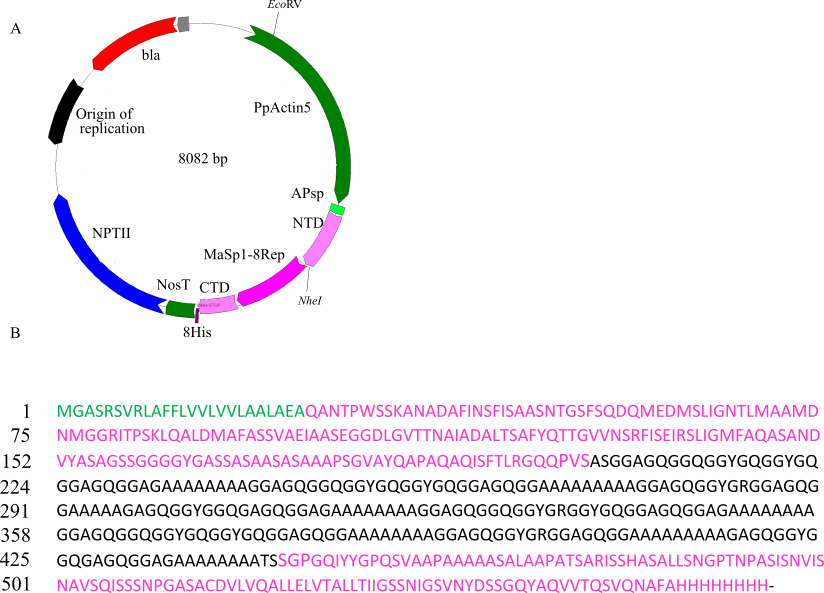


**Fig. S5 Designed construct for the stable production of recombinant NTD-LhMaSp1-8Rep-CTD-8Htag in Physcomitrella. A** Plasmid for the stable NTD-MaSp1-8Rep-CTD**-**8Htag protein production. This plasmid includes the coding sequence of the Physcomitrella Actin5 promoter (PpActin5), APsp, 8Rep, NTD and CTD, 8His-tag, Nos Terminator, nptII (neomycin phosphotransferase) antibiotic-resistance expression cassette, and bla (beta-lactamase). **B** Protein sequence of NTD-LhMaSp1-8Rep-CTD**-**8Htag. Green: Aspartic protease signal peptide (APsp). Black: 8 repetitive Physcomitrella codon-optimized poly-alanine blocks of *L. hesperus* silk protein core region (8Rep). Purple: the full sequence of N and C-terminal domain (NTD and CTD).


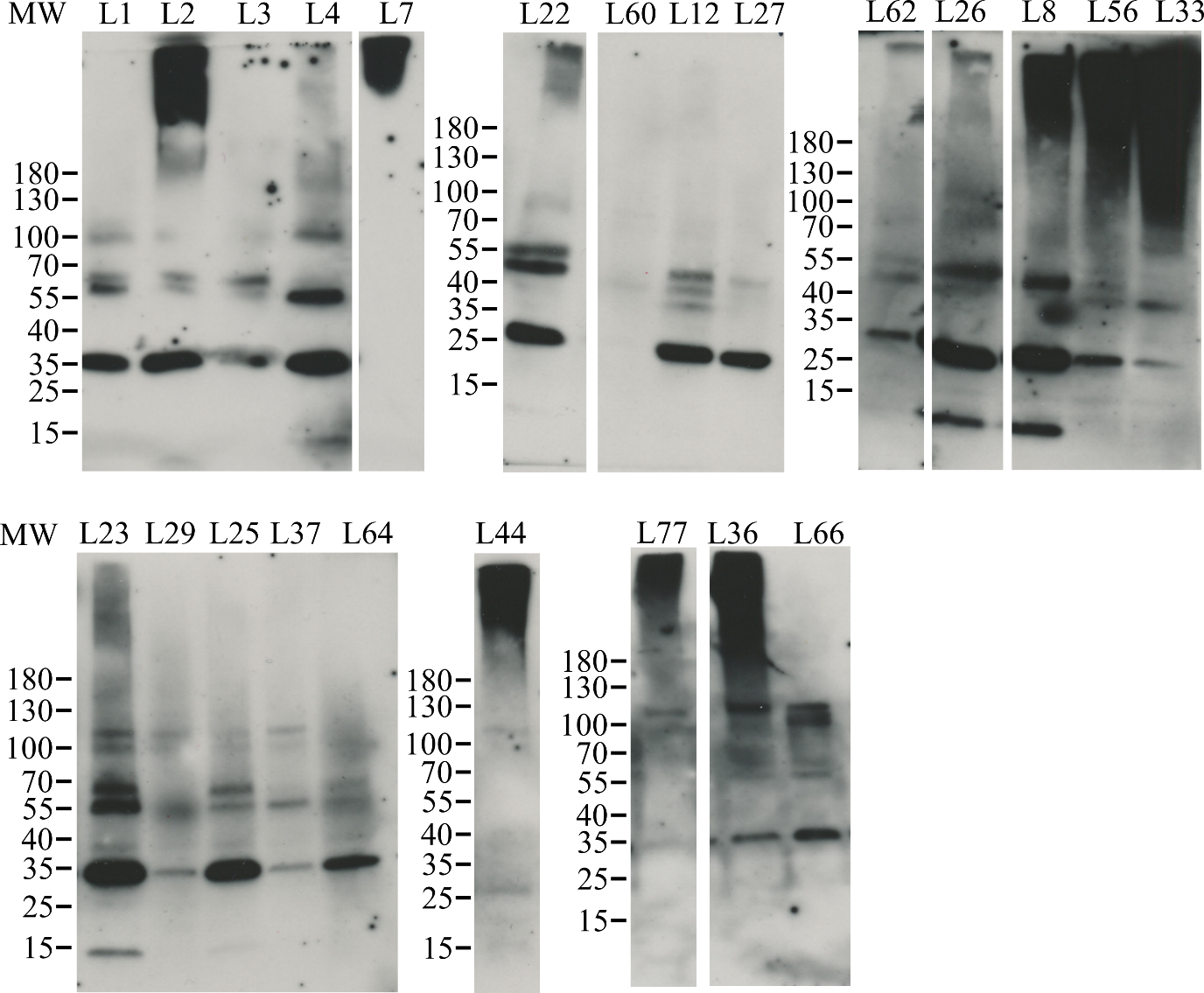


**Fig. S6** **Western blot analysis of all 23 positive lines producing NTD-LhMaSp1-8Rep-CTD-8Htag.** 50 µg of total protein was loaded for each line. Anti His-tag antibody (18184, Thermo Fischer Scientific) was used for detection. L: line. Western blot under reducing conditions. MW: PageRuler Prestained Protein Ladder (Thermo Fisher Scientific).

**
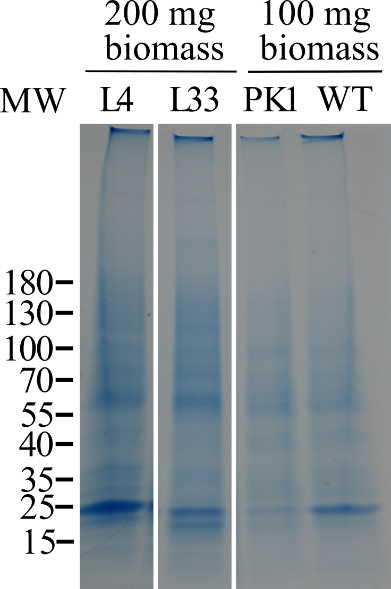
**

**Fig. S7** **Coomassie staining as loading control for the total amount of protein loaded to each lane.** Total soluble proteins extracted from 100 or 200 mg FW were subjected to His SpinTrap column, and 45 µl of the elution of L4, L33, PK1, and WT were loaded onto 4-15% gradient SDS-PAGE gels. PK1: transgenic moss line used as a positive control, as it efficiently produces a 58 kDa protein. L: line. MW: PageRuler Prestained Protein Ladder (Thermo Fisher Scientific).


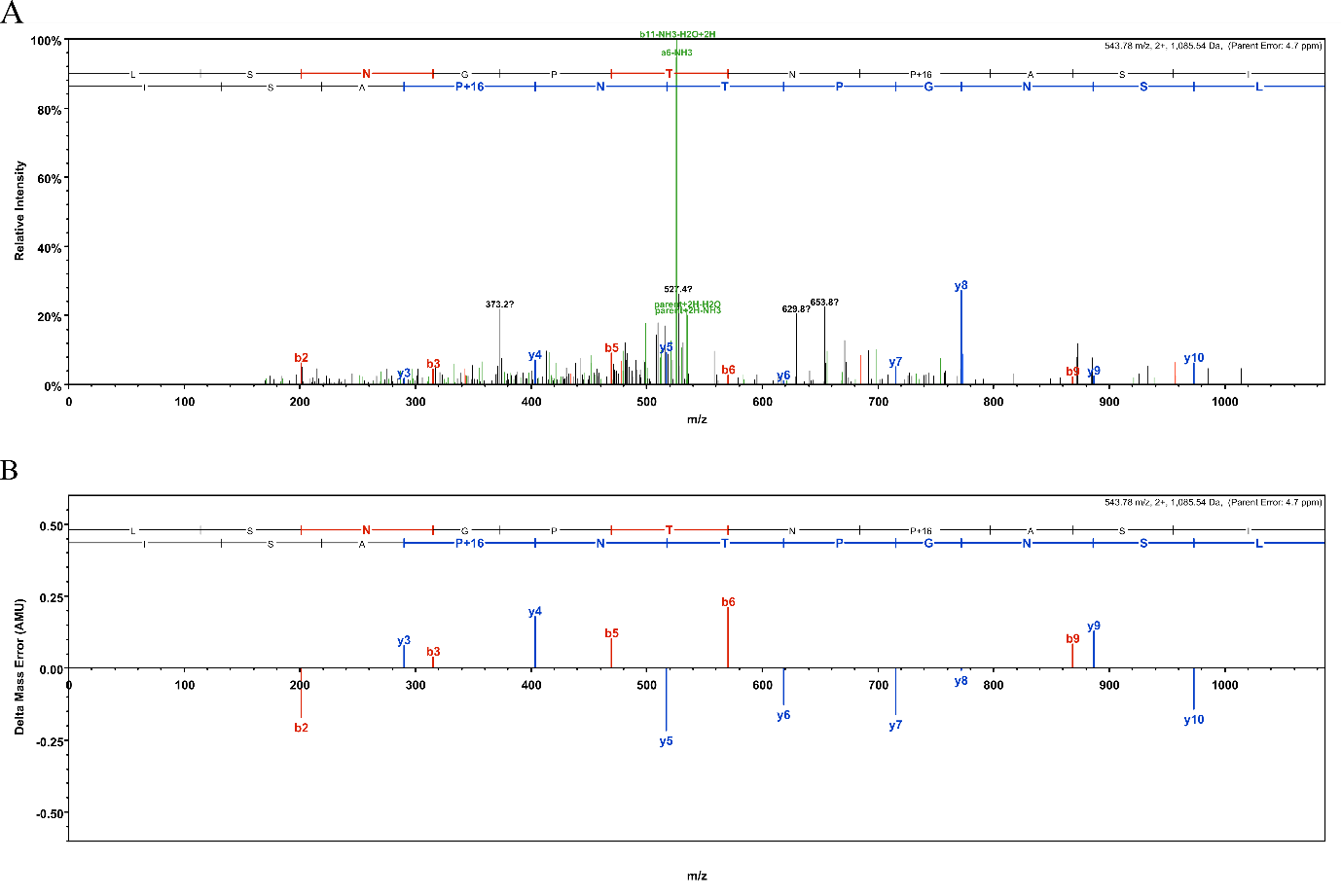


**Fig. S8 Collision-induced dissociation (CID) spectrum and fragment mass error distribution of the prolyl-hydroxylated peptide of MaSp1 recombinantly produced in Physcomitrella.** **A** CID fragment mass spectrum of the peptide LSNGPTN**P**ASI. The mass shift of +16 indicates hydroxylation of the proline. **B** Fragment mass error distribution of b- and y-ions.


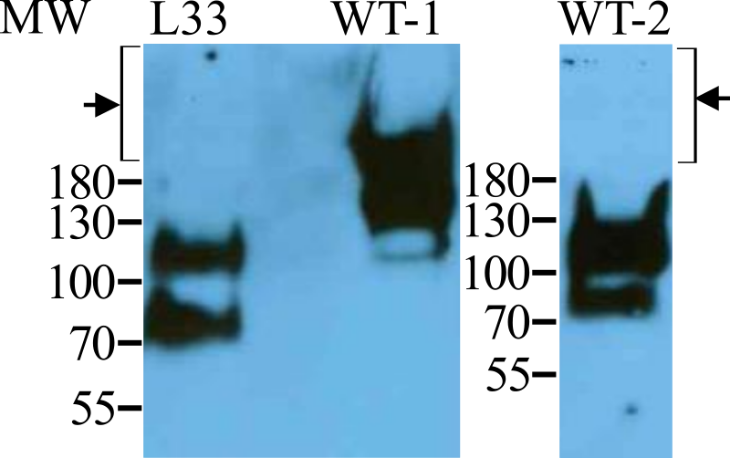


**Fig. S9 Western blot analysis of NTD-LhMaSp1-8Rep-CTD-8Htag, eluted through His SpinTrap column, using JIM 16 antibody against arabinogalactans**. Total extracted proteins from wild-type (WT-1), and proteins of wild-type eluted from the His SpinTrap column (WT-2), were employed as positive controls, for arabinogalactan proteins. The arrow and bracket indicate the absence of arabinogalactans on NTD-LhMaSp1-8Rep-CTD**-**8Htag proteins, as well as negative control. Arabinogalactan proteins are present in the total extracted proteins of WT-1. 10 % SDS gels were used. Western blot under reducing conditions. MW: PageRuler Prestained Protein Ladder (Thermo Fisher Scientific).


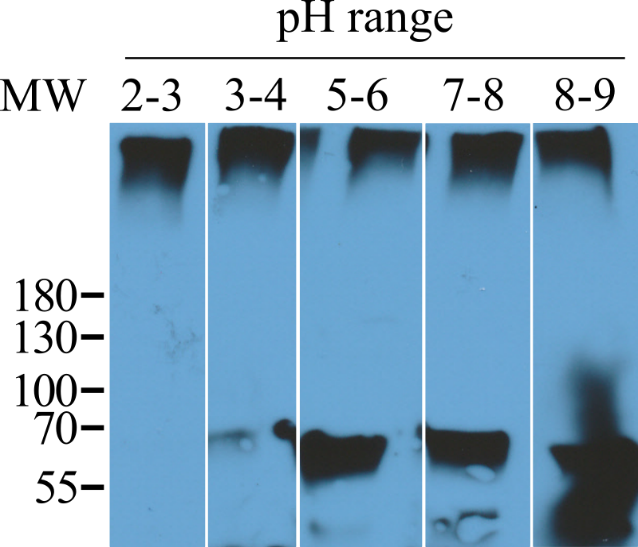


**Fig. S10 Effect of acidic and basic pH levels on the conversion of monomers to multimers and *vice versa***. Variations in pH do not appear to have a significant impact on the conversion of monomers to multimers and *vice versa*. A 10 % SDS gel was used. Western blot under reducing conditions. MW: PageRuler Prestained Protein Ladder (Thermo Fisher Scientific).

**
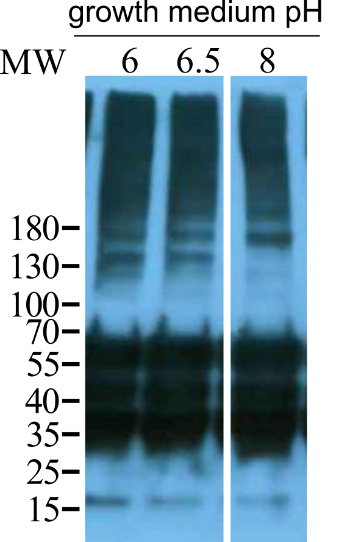
**

**Fig. S11 Effect of different growth medium pH on the high molecular weight signals.** NTD-LhMaSp1-8Rep-CTD**-**8Htag extracted from L33 cultivated in acidic and basic medium with pH 6, 6.5 (buffered with MES), and 8 (adjusted with HEPES buffer), respectively. Western blot analysis showing that variations in the pH of the growth medium does not have any influence on the high molecular weight protein formation. Proteins at 40kDa, 35kDa, and 15kDa are unspecific signals also present in WT samples. MW: PageRuler Prestained Protein Ladder (Thermo Fisher Scientific). A 4-15% gradient SDS gel was used. Western blot under reducing conditions.

**
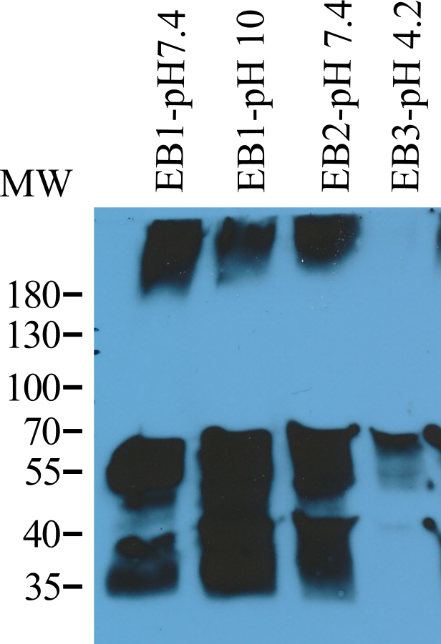
**

**Fig. S12** **Effect of different extraction buffers (EB) and pH values on the high molecular weight signal.** The high molecular weight signal was present for all extraction conditions except for pH 4.2. At pH 4.2, adequate extraction of total protein was not achieved. EB: extraction buffer. EB1: 75 mM HNa_2_PO_4_x2H_2_O_2_ (disodium phosphate), 0.5 M NaCl, 20 mM imidazole, 0.05% Tween-20, and 10% glycerol. EB2: 60 mM Tris, 0.5 M NaCl, 20 mM imidazole, and 10% glycerol. EB3: 60 mM MES, 0.5 M NaCl, 20 mM imidazole, and 10% glycerol. A 10% SDS gel was used. Proteins at 40kDa, and 35kDa are unspecific signals also present in WT samples. Western blot under reducing conditions. MW: PageRuler Prestained Protein Ladder (Thermo Fisher Scientific).


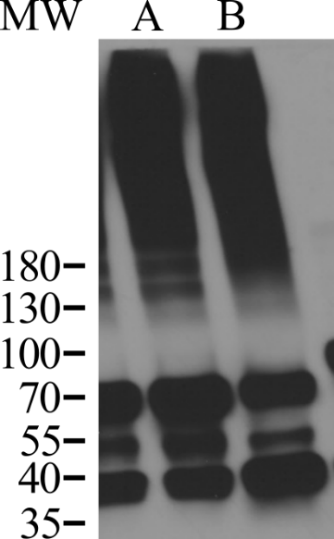


**Fig. S13 Influence of DTT in extraction buffer on the high molecular weight signals of NTD-LhMaSp1-8Rep-CTD-8Htag. A** No DTT in extraction buffer. **B** Adding 75 mM DTT to the extraction buffer did not make a difference on signals from soluble multimer. Western blot under reducing conditions. MW: PageRuler Prestained Protein Ladder (Thermo Fisher Scientific).


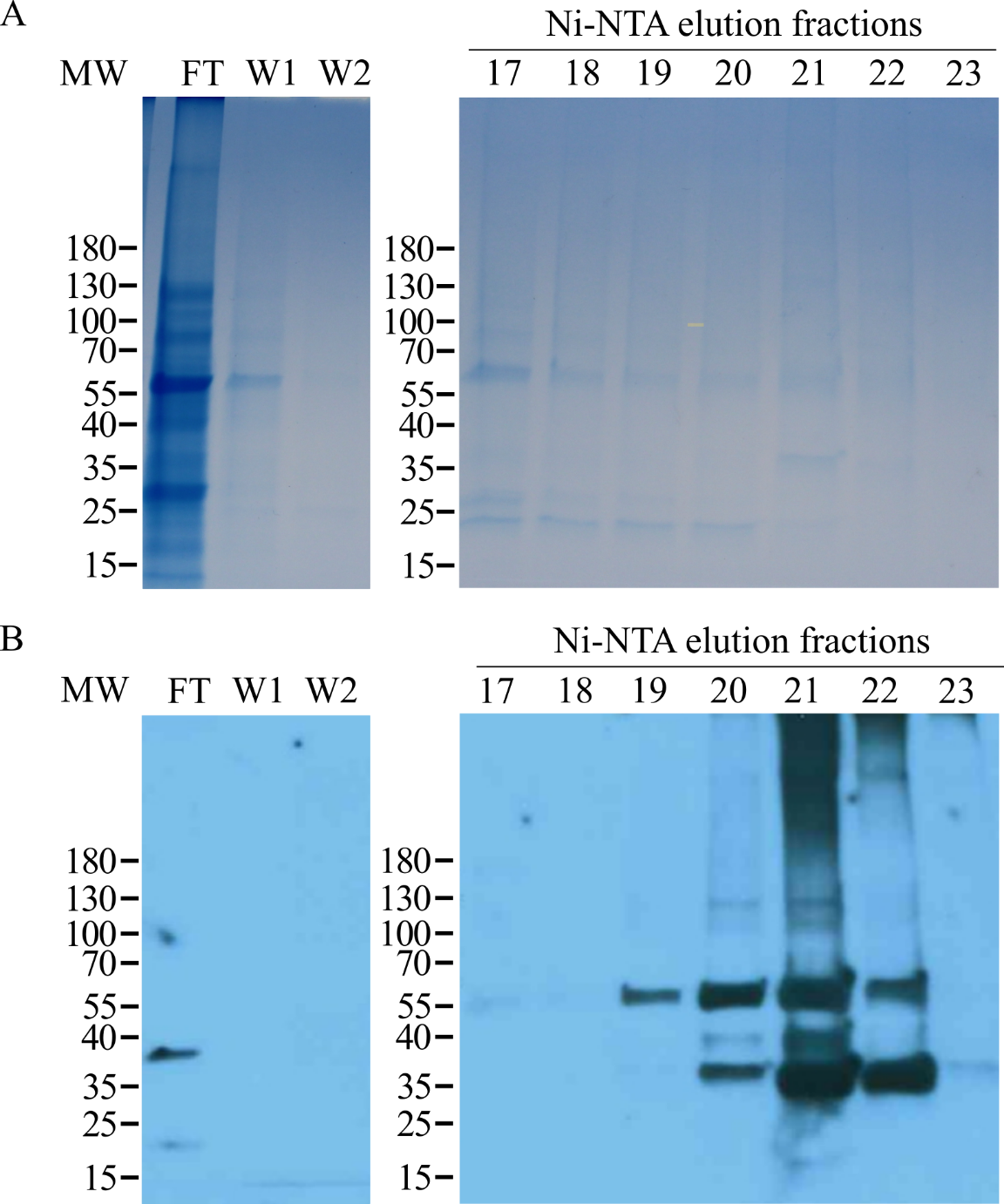


**Fig. S14 Purification of moss-produced NTD-LhMaSp1-8Rep-CTD-8Htag *via* Äkta Ni-NTA chromatography.** **A** Coomassie staining of NTD-LhMaSp1-8Rep-CTD**-**8Htag elution fractions 17-23, Flow-Through (FT), wash1 (W1), and wash2 (W2). **B** Western blot of the same FT, W1, W2, and elution fractions detected with anti-His-tag antibodies. Western blot under reducing conditions. MW: PageRuler Prestained Protein Ladder (Thermo Fisher Scientific). A 4-15% gradient SDS gels were used.

| Name | Sequence | Purpose |
| --- | --- | --- |
| Fwd_overlapAct5_APsp | atattggtggttgttgtgcagaccggtaggcctATGGGGGCATCGAGGAGTG | Gibson assembly |
| Rev_overlapNTD_APsp | gctagaccaaggggtgttcgcctgTGCCTCAGCTAAGGCTGC | Gibson assembly |
| Fwd_overlapAPsp_NTD | gtattagcagccttagctgaggcaCAGGCGAACACCCCTTGGTC | Gibson assembly |
| Rev_overlap8Rep_NTD | cctgtccgccctgcccagctccgccgctagCAGACACAGGCTGCTGTCC | Gibson assembly |
| MaSp1-3m-1684-fwd-CTD | GGACCTGGACAGATCTACTACG | Sequencing |
| MaSp1-3m-278-rev-NTD | GCGATCTCAGCCACGCTA | Sequencing |
| fwd-NTD-firstN | CAGGCGAACACCCCTTGGTCTAGC | Sequencing |
| rev-CTD-lastN | GGCGAAAGCGTTCTGCACGC | Sequencing |
| fwd_NTD_LASTN | GGATACGGTGCATCTAGCG | Sequencing |
| rev_CTD_FIRSTN | ACGTTAGAGATGCTGGCTGG | Sequencing |

**Table S1 Oligonucleotides used for Gibson assembly and sequencing in this work.** Overhangs are shown in lowercase.
